# Increased Plant Quality, Greenhouse Productivity and Energy Efficiency with Broad-Spectrum LED Systems: A Case Study for Thyme (*Thymus vulgaris* L.)

**DOI:** 10.3390/plants10050960

**Published:** 2021-05-12

**Authors:** Jenny Manuela Tabbert, Hartwig Schulz, Andrea Krähmer

**Affiliations:** 1 Plant Analysis and Storage Product Protection, Institute for Ecological Chemistry, Julius Kühn Institute—Federal Research Centre for Cultivated Plants, Königin-Luise-Str. 19, 14195 Berlin, Germany; hs.consulting.map@t-online.de; 2 Institute of Pharmacy, Freie Universität Berlin, Königin-Luise-Str. 2-4, 14195 Berlin, Germany; 3 Consulting & Project Management for Medicinal and Aromatic Plants, Waltraudstraße 4, 14532 Stahnsdorf, Germany

**Keywords:** light-emitting diode, daily light integral, volatile organic compounds, energy consumption, plant morphology, biomass efficacy

## Abstract

A light-emitting diode (LED) system covering plant-receptive wavebands from ultraviolet to far-red radiation (360 to 760 nm, “white” light spectrum) was investigated for greenhouse productions of *Thymus vulgaris* L. Biomass yields and amounts of terpenoids were examined, and the lights’ productivity and electrical efficiency were determined. All results were compared to two conventionally used light fixture types (high-pressure sodium lamps (HPS) and fluorescent lights (FL)) under naturally low irradiation conditions during fall and winter in Berlin, Germany. Under LED, development of *Thymus vulgaris* L. was highly accelerated resulting in distinct fresh yield increases per square meter by 43% and 82.4% compared to HPS and FL, respectively. Dry yields per square meter also increased by 43.1% and 88.6% under LED compared to the HPS and FL lighting systems. While composition of terpenoids remained unaffected, their quantity per gram of leaf dry matter significantly increased under LED and HPS as compared to FL. Further, the power consumption calculations revealed energy savings of 31.3% and 20.1% for LED and FL, respectively, compared to HPS. In conclusion, the implementation of a broad-spectrum LED system has tremendous potential for increasing quantity and quality of *Thymus vulgaris* L. during naturally insufficient light conditions while significantly reducing energy consumption.

## 1. Introduction

Insufficient natural light intensities and short photoperiods drastically limit plant development during winter months in northern regions. Although most common horticultural crops depend on daily light integrals (DLIs) of 6 to 50 mol m^−2^ d^−1^ [1], outdoor solar DLIs often do not exceed 10 mol m^−2^ d^−1^ in higher latitudes during light-limited winter months [2] and are further reduced by up to 60% inside greenhouses [3,4,5]. Therefore, greenhouse industries and research facilities seasonally apply supplemental light sources to prolong cultivation periods and optimize plant growths. However, potentials for (year-round) horticultural productions remain under-utilized, as traditional light sources consume unfeasible amounts of energy [6] and are not tailored to the plants’ photoreceptors [7]. Hence, new technology, which significantly reduces electricity consumption while improving crop value, is of great interest to greenhouse industry and research facilities [8].

Today, light-emitting diodes (LEDs) have the potential to replace traditional light sources such as high-pressure sodium lamps (HPS) [9,10] and fluorescent lights (FL) [11]. They show important technical advantages such as high energy efficiency, small size, durability, long operating lifetime, low thermal emission, and adjustable spectral wavelength range (reviewed in [12,13,14]). Consequently, the utilization of LED technology as horticultural lighting increases [15].

However, the majority of LED radiation studies on plant development have only included narrow wavebands of red (R) and blue (B) light, as these wavelengths are maximally absorbed by the plant’s light-capturing chlorophylls [16]. Initial LED plant-lighting research proved that plants could complete their life cycle with R light alone [17], but the plants’ morphogenesis including compact growth and leaf expansion, as well as plants’ flowering, were significantly improved when differing proportions of B light were included [18,19,20,21,22]. Additionally, specific B light proportions positively influence physiological plant responses such as stomatal opening, chlorophyll contents, and secondary metabolism [8,22].

Recently, studies have suggested further photosynthetic improvements by adding far-red (FR) wavelengths to R spectra, for example, increasing FR radiations promoted growth of seedlings by increasing leaf expansion and whole-plant net assimilation, decreased anthocyanins and carotenoids, and reduced antioxidant potentials [23,24,25].

As recent studies confirm, green (G) light can also contribute to plant development and growth [26,27,28]. Enhanced lettuce growth under RB illumination complemented with G light and improved cucumber growth under HPS supplemented with G light have been reported [29,30,31]. However, G light stimulates early stem elongation and stomatal closure, antagonizing the typical blue-light mediated growth inhibition and stomatal opening [32,33,34].

Due to the multitude of photobiological studies conducted, it is now well established that wavelengths between ~ 360 and 760 nm influence plants’ photosynthesis, physiology, morphogenesis, and phytochemical contents [7] and that specific spectral regions can be used to induce specific plant traits of interest.

Nevertheless, negative side effects resulting from narrow waveband LED applications, such as unwanted photomorphogenic and physiological disorders, pest and disease pressures, as well as difficult visual assessment of plant-status absent under (natural) broad light spectra, have to be further minimized [17,35].

In consequence, LED fixtures with broader spectral quality covering the range of the photosynthetically active radiation (PAR) between 400 and 700 nm (perceived as white light) sometimes including the flanking regions of UV (~360–400 nm) and FR (~600–760 nm) radiation are emerging recently [36] and are becoming popular as sole-source lighting for horticulture [37,38].

For example, Spalholz et al. (2020) compared the response of two lettuce cultivars to a sun-simulated spectrum and other commonly applied B:R spectra, providing a biologically active radiation between 300–800 nm of 200 µmol m^−2^ s^−1^ [39]. The study elucidated unique responses including greatest fresh-to-dry mass ratio, greater leaf area, excessive stem extension, and flower initiation under the sun-simulated spectrum despite a 36% greater photosynthetic photon flux density (PPFD) in B:R treatments. Coinciding results were published by Gao and coworkers (2020), who tested the effects of white and different monochromatic (B, G, Y, R) LEDs on Welsh onions [40]. In addition to increased plant yield, net photosynthetic rate and photosynthetic efficiency were significantly higher under white light than under those of the monochromatic light treatments. Matysiak and Kowalski (2019) observed greatest fresh weights under W and R light treatment for lamb’s lettuce and garden rocket, whereas for two sweet basil cultivars, no differences in fresh weight were detected under all tested light treatments [5]. However, supplementation with B resulted in more compact growth of green-leaved basil. For red pak choi, a white light including UV and FR was evaluated as ideal for best overall yield performance [41], and the importance of white light on shoot and root fresh weights of lettuce was demonstrated [42].

Thus, it has been found that broad LED spectra, covering a wider plant-receptive spectral range rather than single narrow bands, and at best including flanking regions in the FR and UV, can lead to greater plant development. So far, however, such a broad LED spectrum has not been tested under insufficient light conditions in greenhouses. Therefore, our aim was to evaluate the advantages and disadvantages of broadband LED lighting during the winter season in northern central Europe (Berlin, Germany, 52.5° N, 1.33° E) in a practical case study and to compare the results with the common HPS and FL setups found in the greenhouse industry and research facilities today.

As a model plant, we chose moderately light-dependent *Thymus vulgaris* L., which belongs to the Lamiaceae family rich in other genera such as *Salvia* and *Organum* [43] and which is widely used in European cuisine and folk medicine for its expectorant, antitussive, antibroncholitic, antispasmodic, antimicrobial, antioxidant, anti-inflammatory, anthelmintic, carminative, and diuretic properties. The major bio-active metabolite responsible for the therapeutic properties of aromatic *Thymus vulgaris* L. is the monoterpene thymol [44].

The aim of this study was to conduct a greenhouse experiment during winter in order to assess the development, biomass, and health-promoting terpenoid yields of Thymus under a prototype broad-spectrum LED, as well as to obtain the prototypes’ power consumption and efficacy. To further evaluate the practical applicability and potential for greenhouse businesses and research facilities, we aimed at comparing the broad-spectrum LED results with results assessed under HPS and FL fixtures under their common setup conditions.

## 2. Results and Discussion

### 2.1. Biomass Yield, Partitioning, and Morphology

**Figure 1:**
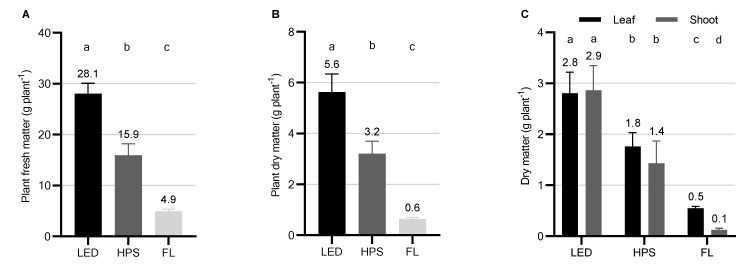
Biomass yields and partitioning of *Thymus vulgaris* L. cultivated under different supplemental lighting systems during fall and winter in Berlin, Germany. LED = light-emitting diode, HPS = high-pressure sodium lamp, FL = fluorescent light. (**A**) Fresh matter yields in gram per plant *, (**B**) Dry matter yields in gram per plant *, (**C**) Leaf and shoot dry matter partitioning in gram per plant **. * Presented are mean plant yields of four independent spatial replications per light treatment (*n* = 4) ± standard deviation (SD) of 32 harvested plants per spatial replication and light treatment (N = 384, *n* = 128 plants per supplemental light treatment, *n* = 32 plants per spatial replication). Significant differences (*p* ≤ 0.01) were determined according to Dunnett’s T3 multiple comparisons test after Brown-Forsythe and Welch ANOVA test (*p* ≤ 0.001). Different letters indicate significant differences. ** Presented are mean dry leaf and shoot matter yields of four independent spatial replications per light treatment (*n* = 4) ± SD (standard deviation) of 16 harvested plants per spatial replication and light treatment (N = 192, *n* = 64 plants per supplemental light treatment, *n* = 16 plants per spatial replication). Significant differences (*p* ≤ 0.05) were determined according to Dunnett’s T3 multiple comparisons test after Brown-Forsythe and Welch ANOVA test (*p* ≤ 0.001). Different letters indicate significant differences.

**Figure 2:**
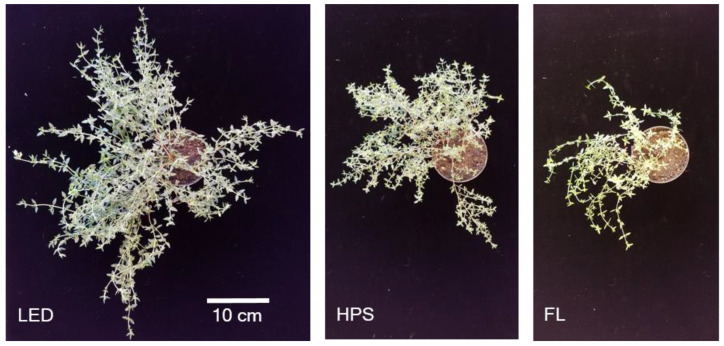
Visual appearance of *Thymus vulgaris* L. at harvest cultivated under different supplemental lighting systems during fall and winter of Berlin, Germany. LED = light-emitting diode, HPS = high-pressure sodium lamp, FL = fluorescent light.

The LED system resulted in distinct yield increases, biomass partitioning, and a differentiated morphological appearance of *Thymus vulgaris* L. in comparison to the HPS and FL systems (Figure 1 and Figure 2). While the LEDs produced a fresh biomass of averagely 28.1 ± 2.0 g plant^−1^, the HPS systems accounted for a fresh biomass of 15.9 ± 2.3 g plant^−1^ within the same cultivation period. The lowest fresh biomass of 4.9 ± 0.4 g plant^−1^ was produced under FL (Figure 1A). Accordingly, dry matter yields of *Thymus vulgaris* L. were significantly enhanced by the LED system (5.6 ± 0.8 g plant^−1^) in comparison to HPS (3.2 ± 0.5 g plant^−1^) and FL (0.6 ± 0.1 g plant^−1^), representing an increase of 1.75- and eight-fold, respectively (Figure 1B). Thereby, the weight proportion of dry leaves did not differ from the (mostly lignified) weight proportion of stems in thyme plants cultivated under the LED and HPS systems, respectively. Under FL, however, the majority of dry yield consisted of leaves (83.3%) and only 16.7% consisted of (unwooded) shoots (Figure 1C). With a Pearson correlation coefficient of r = 0.97 and R^2^ = 0.95 (*p* < 0.001), dry mass yields under the differing supplemental lighting systems were highly related to the individual daily light integrals (DLI).

As indicated in Figure 2, the stem biomass of *Thymus vulgaris* L. was greatly increased under the LED system at the end of the experimental period and led to a profoundly different visual appearance in comparison to thyme plants grown under the other two supplemental lighting fixtures. Despite the lowest corresponding leaf-to-shoot ratio, which was 0.9 for LED, 1.3 for HPS, and 5 for FL, the leaf dry matter (L_DM_) of thyme was significantly increased and highest under LED (Figure 1C).

The reason for the outstanding biomass accumulations and the concomitant rapid thyme development under the LED system is clearly found in the heightened DLI between 400 and 700 nm, as shown by the correlation coefficient of r = 0.97 (R^2^ = 0.95). That increasing DLIs accelerate the development and growth of plants up to a certain point is well established [45,46]. The correlation of DLIs and plant growth is known to be linear between each species-specific light compensation point and light saturation point [7].

Faust stated that optimal DLIs vary from 6 to 50 mol m^−2^ d^−1^ for various crops, and moderately light-dependent thyme requires a DLI of at least 18 mol m^−2^ d^−1^ [46]. The natural average DLI in greenhouses during winter in northern latitudes however is often as low as 1 to 5 mol m^−2^ d^−1^ and reached approximately 3.9 mol m^−2^ d^−1^ during our greenhouse trial [3,4,5]. Hence, supplemental lighting is essential for winter greenhouse productions. Since FLs raised the total DLI (natural DLI 3.9 mol m^−2^ d^−1^ + supplemental DLI 3 mol m^−2^ d^−1^) only to approximately 7 mol m^−2^ d^−1^ during winter production, the FLs are neither suitable for the production of thyme nor presumably for the majority of greenhouse crops under the given cultivation conditions. HPS elevated the total DLI (natural DLI 3.9 mol m^−2^ d^−1^ + supplemental DLI 7 mol m^−2^ d^−1^) to an estimated level of 11 mol m^−2^ d^−1^. Therewith, the biomass accumulation of *Thymus vulgaris* L. increased significantly in comparison to FL; however, the DLI remains insufficient for an optimal thyme production during winter. With a total DLI of approximately 16 mol m^−2^ d^−1^ (natural DLI 3.9 mol m^−2^ d^−1^ + supplemental DLI 11 mol m^−2^ d^−1^) during the low light season, the tested LED system achieved the highest DLI and approached the recommended DLI of ≥ 18 mol m^−2^ d^−1^ the most (Table 1, Section 3. Material and Methods). Further, only the LED system would be able to achieve the recommended DLI of the moderately light-dependent thyme by simply extending the photoperiod from 14 to 16 h per day during winter.

### 2.2. Content and Composition of Volatile Organic Compounds (VOCs)

Applying GC-MS analysis, 12 monoterpenes and one sesquiterpene were identified in the leaf extracts of *Thymus vulgaris* L., representing ≥ 94% of all detected volatile constituents. Major identified volatile organic compounds (VOCs) in the leaf extracts of *Thymus vulgaris* L. under the supplemental lighting systems were thymol, *γ*-terpinene, and *p*-cymene, respectively, which is consistent with the results of former research [48,49]. Thereby, the chemical makeup remained unaffected by the different lighting systems. The total content of VOCs per g of L_DM_ is highly enhanced by LED (2.7%) and HPS (2.3%) as compared to by FL (1.1%). The difference in quantity of VOCs per g of L_DM_ between thyme plants cultivated under LED and HPS is not significant (*p* = 0.088). The LED considerably increased the amounts of all 13 evaluated terpenoids in the leaves of *Thymus vulgaris* L. in contrast to the FL system. The HPS system also enabled considerable increases in comparison to the FL system, even though the differences between the amounts of *γ*-terpinene and borneol are less profound, with *p* = 0.099 and *p* = 0.075, respectively. Differences between LED and HPS treatments were only detected for *α*-pinene, while myrcene (*p* = 0.077) as well as limonene (*p* = 0.057) differed only in tendency. All results are summarized in Table 2.

Gouinguene and Turlings (2002) showed in their study that young corn plants (*Zea mays* L.) significantly increased their emissions of volatiles as light intensity increased up to 10,000 lm [50]. However, beyond 10,000 lm volatile emissions in *Zea mays* L. did not enhance any further, suggesting a kind of saturation or limitation was reached. The authors of [51,52] also detected this proposed light quantity-dependency. Multiple studies also suggest that terpene synthesis involves phytochromes [51,52,53,54], red and far-red light-sensing photoreceptors (reviewed by [55]), making the production of terpenes also dependent on light quality, specifically on the *R/FR* ratio [56]. For example, in thyme seedlings, red light strongly promoted the production of mono- and sesquiterpenes (thymol, *γ*-terpinene, *p*-cymene and carvacrol, *β*-caryophyllene) and the number of essential oil-containing trichomes per cotyledone, two stimulatory effects that proved to be completely reversible by a subsequent exposure to far-red irradiation [53,54]. Later, a partial reduction of volatile emissions was detected in *Arabidopsis thaliana* (L.) Heynh exposed to a low *R/FR* ratio of 0.2 as compared to plants exposed to a high *R/FR* ratio of 2.2 when controlling for light intensity [56]. These findings could explain the comparatively low VOC contents detected in thyme leaves grown under FL, as both their light intensity (*PFD* 60 µmol m^−2^ s^−1^, *PFD*-R 7.6 µmol m^−2^ s^−1^) as well as their *R/FR* ratio (0.1) were significantly reduced as compared to LED and HPS under our experimental conditions. It would also suggest that the similar contents of VOCs per gram of thyme leaves found under LED and HPS are the result of their similar high *R/FR* ratios of 2.8 and 2.4, respectively. However, *R/FR* ratios dramatically decline under vegetational canopies. As described by Franklin (2008), a single leaf reduces a given *R/FR* ratio of 1.2 to 0.2 for the leaves growing underneath, and the ratio reduces further to 0.1 underneath a second leaf [55]. As leaf and shoot yields of *Thymus vulgaris* L. significantly increased under the LED system, it is reasonable to believe that the actual *R/FR* ratio underneath the more densely stands of thyme plants grown under the LED system was much lower than under the less dense canopy of thyme plants grown under HPS. This idea coincides with the result from Kegge et al. (2013), who detected a reduction of volatile emissions in plants grown in high density stands [56]. Another explanation for the similar contents of VOCs per gram of thyme leaves found under LED and HPS may be found in the high B light proportion found under the broad LED light spectrum, as it was recently shown that essential oil contents of *Thymus vulgaris* L. decrease with increasing proportions of blue light [57]. The associated suppressions of terpene synthesis under low *R/FR* ratios as well as under low *R/B* ratios may have been partially compensated by the LEDs’ elevated light intensity (*PFD* 232 µmol m^−2^ s^−1^, *PFD*-R 55.3 µmol m^−2^ s^−1^) compared to the intensity of the HPS system (*PFD* 143 µmol m^−2^ s^−1^, *PFD*-R 24.3 µmol m^−2^ s^−1^) in our study. Additionally, though air temperatures under the given experimental conditions did not differ between the supplemental lighting systems, it is known that leaf temperature increases under HPS lights as comparted to other lighting systems [58]. As elevated temperatures evidently increase the emission of volatiles [50], a greater leaf temperature under HPS may have been present and contributed to the terpene synthesis in HPS-grown thyme plants. Further, as we did not adjust fertilization, though the LED system yielded much greater biomasses than FL and HPS, it is plausible that a reduced nutrient availability for LED-grown thyme plants limited their production of VOCs, as demonstrated by Gouinguene, and Turlings (2002), who showed that fertilization rate positively effects volatile emissions [50].

Nevertheless, as the LED lights were able to increase the production of volatiles in thyme leaves significantly compared to the HPS lights, the LEDs’ volatile productivity per square meter doubled in absolute terms (2.5 vs. 1.3 g m^−2^) with *p* < 0.06 (Table 3).

### 2.3. Productivity

The broad-spectrum LED system enabled a highly significant increase in leaf and stem production of fresh thyme per square meter, representing increases of 43.3% and 82.4% in comparison to the HPS and FL system, respectively. Additionally, the dry matter productions of HPS and FL were highly reduced by 43.1% and 88.6% in comparison to the LED system. Further, the LED system enabled an increase in production of VOCs per square meter under the given greenhouse conditions in comparison to the conventionally used HPS system (at *p*-value of 0.051). Both systems (LED and HPS) considerably promoted the VOC production in comparison to the FL system. Table 3 summarizes the results. Despite the lower leaf-to-shoot ratio for LED lightning of 0.9 as compared to both HPS (1.3) and FL (5, see Section 2.1), absolute L_DM_ and overall quantity of VOCs were highest for LED. Therefore, LED lightning offers an attractive alternative for thyme cultivation, both for essential oil production and delivery to fresh market.

### 2.4. Power Consumption and Biomass Efficiacy

The LEDs consumed the least electricity with 257.7 W m^−2^, followed by the FLs with the use of 299.4 W m^−2^, whereas the HPS lamp consumed the highest amount of electricity with 374.9 W m^−2^. At the end of the cultivation period, the power consumptions per m^2^ of LED and FL lighting system resulted in high energy savings of 31.3% and 20.1%, respectively, when compared to the consumption of the HPS system. While each LED system enabled ± 1.92 g of fresh thyme per kWh and square meter, the HPS and FL enabled only ± 40% and ± 16% of these yields per kWh and square meter, respectively. Accordingly, the dry thyme production per kWh and square meter under LED (±396.3 mg) was significantly higher than the dry thyme production under HPS (±155.2 mg) and FL (±39.1 mg). Further, the production of VOCs per kWh and square meter was significantly elevated underneath the LEDs (±5.4 mg) as compared to HPS (±1.9 mg) and FL (±0.4 mg). Results and calculations are combined in Table 4.

Our power consumption results and thus the potential of LEDs for reducing energy costs coincide with numerous studies and reviews [6,9,59], stating energy reductions up to 70% compared to traditional light sources while producing similar crop yields at equal light intensities, and confirm the current trend of LEDs’ increasing photon efficiencies: While HPS and LED fixtures had nearly identical photon efficiencies until ~2015 [6,35], the best evaluated LED fixture was 40% more photon-efficient than HPS due to technological improvements of LEDs within the PAR region soon after [35,59]. A current study by Hernandez et al. (2020) confirms the corresponding increase in biomass efficacy of LEDs, as their LED treatment led to a 2.4 to 3.1 times greater biomass efficacy than HPS, which matches our findings [60]. Another study in which LED and FL treatments were compared, reported a biomass efficacy three to five times higher under LED than under FL lighting [61]. In contrast, the LED system used in this current study greatly exceeds their findings, as the LED enabled a biomass efficacy 6 to 10 times higher than the FL system (Table 4) under our experimental conditions.

**Figure 3:**
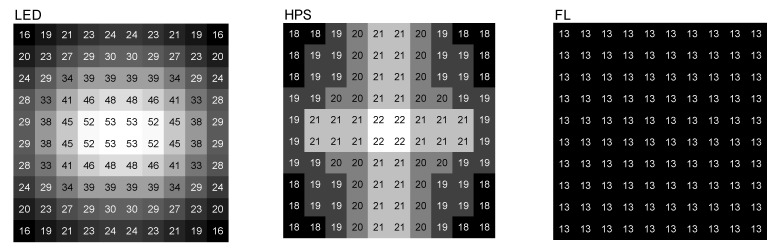
Irradiance profiles (W m^−2^ nm^−1^) of the experimental plots (1 m^2^) underneath each supplemental lighting system. (LED = light-emitting diode, HPS = high-pressure sodium lamp, FL = fluorescent light).

Further, in our study, plant growth may have been limited by nutrient availability, and an adjustment of fertilization based on the differing thyme growth rates may further increase biomass efficacies under HPS and especially under the broad-spectrum LED system. Nevertheless, when using the broad-spectrum LED lighting system, the significantly more inhomogeneous light intensity distribution compared to HPS and FL lamps (Figure 3) must be taken into account when light uniformity is necessary for the greenhouse application as it demands more LED light fixtures per area.

Nevertheless, at their edges, where the lowest light intensities occur, the LEDs achieve values of 16 W m^−2^ nm^−1^, which are sufficient for high-quality thyme production. Therefore, if homogeneous plant development is not necessarily required, plants of marketable quality are also available with the LED setup used here without additional lamps.

## 3. Materials and Methods

### 3.1. Experimental Design

To investigate biomass yields and contents of volatile organic compounds (VOCs) of *Thymus vulgaris* L. grown under a broad-spectrum LED system, and to compare the lights’ productivity as well as electrical efficiency with conventionally used lighting fixtures for the cultivation of thyme under naturally low irradiated greenhouse conditions during fall and winter in Berlin, Germany, a one-factorial experiment with a randomized block design with three different supplemental light sources and four spatially independent replications (N = 384; *n* = 32 thyme plants per replication) was conducted.

### 3.2. Lighting Systems and Illumination Conditions

Three different supplemental light sources ((1) fan-cooled light-emitting diode (LED) (SUNtec Technology, FUTURELED^®^, Berlin, Germany, dimensioning 47.5 × 21.5 × 19.5 cm^3^), (2) high-pressure sodium (HPS) lamps (bulb: SON GreenPower CG T 400 W E40 1SL, PHILIPS, Hamburg, Germany; ballast: HST, SILL Leuchten^®^, Berlin, Germany, dimensioning 50 × 30 × 19 cm^3^), and (3) fluorescent lamps (FL) (VENEDIG, Pracht^®^, Berlin, Germany, dimensioning 50 × 50 × 16 cm^3^)) were horizontally mounted onto given steel frames 1.40 m above greenhouse benches, resulting in distances between the bottom of the LED, HPS, and FL light sources and the greenhouse benches of 1.14, 1.13, and 1.09 m, respectively. Based on weather recordings from WetterKontor [62], plants were exposed to an average of 2.5 h of sunshine per day during the experiment. In addition to the natural sunlight, plants were subject to supplemental lighting from 6:00 a.m. to 8:00 p.m. for a photoperiod of 14 h per day during the greenhouse experiment. Plastic sheeting extending from above the light fixtures to below the greenhouse benches eliminated neighboring light pollution.

### 3.3. Irradiance Profile Measurements

**Figure 4:**
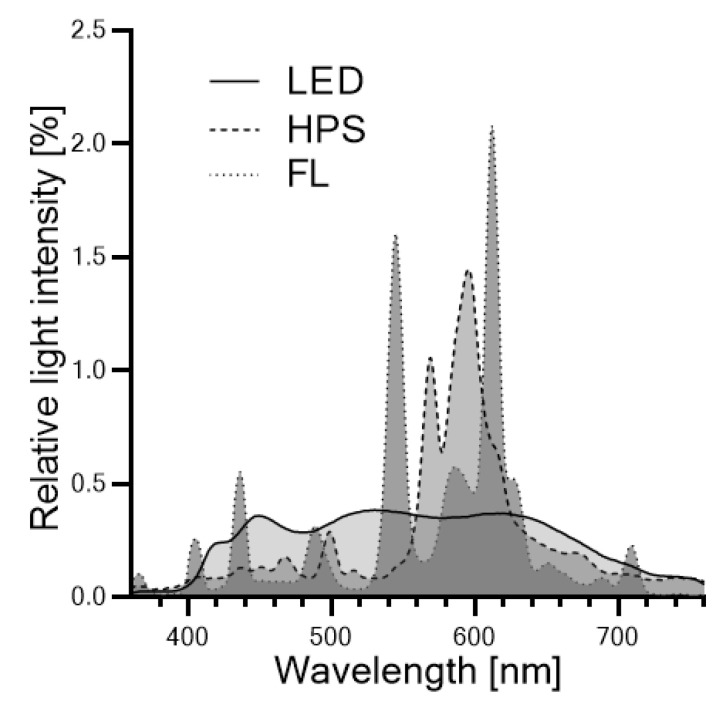
Light spectra of the three artificial light sources (light-emitting diode (LED) = solid line, high-pressure sodium lamp (HPS) = dashed line, fluorescent light (FL) = dotted line) used during the greenhouse experiment.

Irradiance measurements of the light fixtures were taken prior to the experiment using a spectral PAR meter (PG200N, UPRtek, Aachen, Germany) at night. Light intensity, spectral composition, and irradiance profiles (light distribution patterns) were measured and recorded at bench level under experimental conditions. The software package of the spectrometer (uSpectrum PC laboratory software) automatically calculated all electromagnetic parameters including photon flux density (PFD in µmol m^−2^ s^−1^) and spectral irradiance (W m^−2^ nm^−1^) between 360 and 760 nm and photosynthetic photon flux density (PPFD in µmol m^−2^ s^−1^) between 400 and 700 nm. During a sunny day, light transmission between 350 and 800 nm of the natural irradiance through the greenhouse glass was determined to be 28% (±5%) by comparing the output of the spectrometer inside the greenhouse with the output outside the greenhouse and resulted in a photon flux density of ~434 µmol m^−2^ s^−1^ at bench level, which amounts to an approximate natural daily light integral of 3.9 mol m^−2^ d^−1^ when combined with the weather recordings (Section 3.2). Light spectra and detailed spectral compositions of the supplemental lighting systems are depicted in Figure 4 and summarized in Table 1, respectively. Under each lighting system, the irradiance profiles within one square meter (representing the replicated experimental plots during the greenhouse experiment) were measured over a flat plane below the fixtures at intervals of 10 cm between 360 and 760 nm. Each measurement represents the spectral irradiance in W m^−2^ nm^−1^ and was replicated three times and averaged, leading to a total dataset of 100 measurements per square meter. These irradiance profiles are depicted in Figure 3.

### 3.4. Plant Material and Growth Conditions

Seeds of *Thymus vulgaris* L. (Rühlemann’s Kräuter- und Duftpflanzen, Horstedt, Germany) were sown in 128-cell plug trays (Ø 4 cm) filled with potting substrate (Fruhstorfer Einheitserde Typ P, HAWITA, Vechta, Germany) on 9 October 2018 and placed under the differing lighting fixtures in a NS-orientated greenhouse located in Berlin, Germany (52.5° N, 13.3° E). After six weeks on 20 November 2018, 32 representative seedlings were transplanted into pots (Ø 9 cm) containing substrate with an elevated nutrient composition (Fruhstorfer Einheitserde Typ T, HAWITA, Vechta, Germany) and evenly placed (quadratic) within 1 m^2^ under each light fixture. Each treatment was replicated four times, positioned in a randomized block design, and surrounded by 28 border plants to avoid boundary effects. Starting 27 December 2018, plants were fertilized weekly with 100 mL of a 0.2% (*v*/*v*) nutrient solution (Hakaphos^®^ Blau, COMPO EXPERT^®^, Muenster, Germany) containing 15% N, 10% P_2_O_5_, 15% K_2_O, 2% MgO, 0.01% B, 0.02% Cu, 0.075% Fe, 0.05% Mn, 0.001% Mo, and 0.015% Zn. However, due to the low biomass accumulation of the plants grown under the fluorescent lamp system, the plant fertilization was started two weeks later under the FL treatment. All thyme plants grew for a total period of 18 weeks until harvest on 12 February 2019. Climatic conditions including temperature and relative humidity of the greenhouse air were continuously monitored at canopy level via data loggers (EL-USB−2, Lascar, CONRAD, Hirschau, Germany). Average temperatures (°C ± SD) under LED, HPS, and FL lighting were 20.4 ± 1.4, 21.1 ± 2.0, and 20.9 ± 1.2, with a measuring accuracy of 1 °C. Average humidities (%rh ± SD) under LED, HPS, and FL lighting were 47.2 ± 7.0, 44.2 ± 7.1, and 38.8 ± 7.1 with a measuring accuracy of 2.25%rh. Both climatic conditions did not differ between treatments.

### 3.5. Harvest and Crop Managements

To analyze the effect of the supplemental lighting systems on the yield, all 32 experimental thyme plants were harvested separately from each treatment condition and replication. Fresh matter (FM) of the above-ground plant parts was individually recorded at harvest on 12 February 2019. Total dry matter (DM) was measured after drying the samples in a circulated drying oven at 30 °C until stable mass was attained (≤seven days). Leaf dry matter (L_DM_) was determined for 16 plants selected from each treatment and replication, and the corresponding shoot dry matter (S_DM_) was calculated by subtracting the LDM from DM. All dried leaf samples were vacuum-sealed (V.300^®^, Landig + Lava GmbH & Co KG, Bad Saulgau, Germany) and stored in the dark at 4 °C until further processing.

### 3.6. Energy Measurements

The power draw of current (I) and voltage (U) as well as electrical characteristics including real power (P) and apparent power (S) from representative lamps of each lighting treatment were measured using a power meter (ENERGY MONITOR 3000, VOLTCRAFT^®^, Wernberg-Köblitz, Germany) in order to estimate energy consumptions and biomass efficacies of the light fixtures. To correct for the detected difference between P and S due to heat dissipation of the HPS system, the measured *cos phi* of 0.93 was incorporated into the HPS’ power consumption calculations. According to the manufacturer’s specifications, the HPS system allows a homogeneously illuminated area of 1.56 m^2^ (1.2 × 1.3 m). Thus, the measured power consumptions were adjusted to the power consumption per square meter (W m^−2^) via rule of three. No adjustments were necessary for the LED and FL system.

### 3.7. Chemicals

Pure standard substances (*α*-pinene, *α*- and *γ*-terpinene, *β*-caryophyllene, borneol, carvacrol, limonene, linalool, myrcene, *p*-cymene, sabinene, thymol, and 6-methyl-5-penten-2-one) were purchased as analytical standards with a purity of at least 95% for GC reference analysis from Alfa Aesar (Kandel, Germany), Carl Roth (Karlsruhe, Germany), Merck KgaA (Darmstadt, Germany), and Fluka (Seelze, Germany). Isooctane (> 99%, HPLC grade) for solvent extraction of volatile organic compounds was obtained from Th. Geyer (Renningen, Germany).

### 3.8. Extraction of Volatile Organic Compounds

The volatile organic compounds (VOCs) of 16 plants per treatment and replication (*n* = 64) were extracted according to the following procedure: 100 mg (±2%) of gently oven-dried and powdered (3 intervals of 10 s at 15,000 rpm via Tube Mill control, IKA^®^, Staufen, Germany) thyme leaves were transferred into 2 mL screw cap micro tubes (SARSTEDT AG & Co. KG, Nümbrecht, Germany) including two steal grinding balls (Ø 2 mm). The plant material was homogenized in 1.0 mL of isooctane (containing 1:40,000 (*v*/*v*) 6-methyl-5-penten-2-one as internal standard) for 10 min at 30 rps with a ball mill (MM400, Retsch^®^, Haan, Germany). After 10 min of ultra-sonication (Sonorex RK 106, BANDELIN electronic GmbH & Co. KG, Berlin, Germany) and 10 min of centrifugation at 13,000 rpm (Heraeus™ Labofuge™ 400 R, Thermo Scientific™, Osterode, Germany) at 22 °C respectively, the supernatants were transferred into GC-vials and stored at −70 °C until analysis.

### 3.9. GC-FID and GC-MS Analysis

A total of 1 µL of the obtained extracts of volatiles was analyzed by GC–FID using an Agilent gas chromatograph 6890N fitted with an HP-5MS column (30 m × 250 µm × 0.5 μm) in splitless mode. Detector and injector temperatures were set to 250 °C. The following oven temperature program was used: 50 °C for 2 min, heating from 50 to 320 °C at a rate of 5 K min^−1^. The final temperature was held for 6 min. Hydrogen was used as carrier gas with a constant flow rate of 1.2 mL min^−1^. GC-MS was performed using an Agilent mass spectrometer 5975B, on an HP-5MS column (see GC), operating at 70 eV ionization energy, using the same temperature program as above. Helium was used as carrier gas with a constant flow rate of 1.2 mL min^−1^. Retention indices were calculated by using retention times of C_6_-C_24_-alkanes that were injected under the same chromatographic conditions.

### 3.10. Identification and Quantification of Volatile Organic Compounds

All main organic compounds of the volatile extracts were identified by comparing their mass spectra with those of internal reference libraries (Adams, NIST). Additionally, the identification of *α*-pinene, *α*- and *γ*-terpinene, *β*-caryophyllene, borneol, carvacrol, limonene, linalool, myrcene, *p*-cymene, sabinene, and thymol was confirmed by authentic reference standards by comparing their individual retention indices. Quantitative data of each main compound were obtained with serial dilutions of external standard solutions using at least six known concentrations (*β*-caryophyllene, *cis*-sabinene hydrate and linalool: 0.5, 1, 2, 5, 10, and 50 ng µL^−1^; *α*-pinene, *α*-terpinene, borneol, limonene, myrcene, and sabinene: 1, 2, 5, 10, 50, and 100 ng µL^−1^; *p*-cymene: 1, 2, 5, 10, 50, 100, 200, and 400 ng µL^−1^; *γ*-terpinene: 1, 2, 5, 10, 50, 100, 200, 400, and 600 ng µL^−1^; carvacrol: 1, 2, 5 10, 50, 100, 200, 400, 600, and 1200 ng µL^−1^; thymol: 5, 10, 50, 100, 200, 400, 600, 1200, and 1800 ng µL^−1^), which covered concentration ranges detected for each compound in all samples.

### 3.11. Statistical Analysis and Calculations

Statistical analysis was performed using GraphPad Prism 8.4.2.679 (San Diego, MO, USA). Data of each spatial replication were tested for normality via Anderson-Darling, D’Agostino and Pearson, Shapiro–Wilk test, and Kolmogorov–Smirnov test. If normality test failed, outliers were identified via ROUT method (Q = 10%) and removed to establish normality of all data sets. The means of each spatial replication (*n* = 4) were tested for normality with the Shapiro–Wilk test, and all data sets passed the normality test at α = 0.05. Finally, all data sets were statistically analyzed using a Brown-Forsythe and Welch ANOVA test due to unequal variances between treatments. Multiple comparisons were conducted via Dunnett T3. Two-tailed Pearson correlation between biomasses and DLIs was calculated after first computing means of 18 replicates per spatially independent light treatment and then analyzing those means (*n* = 12). Biomass efficacies (g or mg kWh^−1^ m^2^) were calculated based on electricity consumed within a square meter during the cultivation period.

## 4. Conclusions

With its outstanding biomass as well as terpenoid efficacy, the broad-spectrum LED system represents a strong competitor to the conventionally used HPS and FL lighting systems in greenhouses under naturally insufficient light conditions as investigated in this study. Marketable *Thymus vulgaris* L. can be achieved faster and thus more often when replacing HPS and FL light sources with the tested LED system, ultimately resulting in greater revenues at simultaneously highly reduced production costs for greenhouse growers. The comparatively high initial capital costs of LEDs which have delayed their establishment in the past are decreasing [59]. Based on our results, combined with the typically low maintenance and long operating lifetime [13,15,63,64], the initial investment into LEDs should quickly become a source of profit for greenhouse growers. Additionally, different adaptive control approaches making use of the dimmability of LEDs [35,65] can further decrease the power consumptions and help to achieve consistent growth rates at a daily and seasonal level as shown by [60,66]. Our results suggest that an implementation of a broad-spectrum LED system in greenhouses could provide the possibility to cultivate a greater variety of crops with greater DLI-requirements under naturally insufficient light levels as conventional lighting systems are capable of today. Further, the broad-spectrum LED system could extend greenhouse production seasons, which are currently constrained by low supplemental DLIs, and allow a year-round production of a wider variety of selected greenhouse crops then HPS and FL systems are able to at present. However, further trails with a variety of greenhouse crops need to be investigated to confirm the suggested applicability for a range of crops. Therefore, in prospective broad-spectrum LED studies, the crops’ individual DLI requirements need to be incorporated and compared to commonly applied mono- and dichromatic LED light spectra at equal light intensities for advancing our knowledge on the impact of LED light spectra on morphological, physiological, and metabolic plant responses. In addition, more studies examining the impact of light qualities on terpenoid biosynthesis, content, and composition are needed to optimize the quality of aromatic plant species in the future.

## Figures and Tables

**Table 1:** Spectral composition of the supplemental lighting fixtures used in the greenhouse for the cultivation of thyme (*Thymus vulgaris* L.).

Parameter *	Supplemental Light Fixtures **					
	LED		HPS		FL	
	µmol m^−2^ s^−1^	% ***	µmol m^−2^ s^−1^	% ***	µmol m^−2^ s^−1^	% ***
PPFD (400–700 nm)	212	91.2	132	92.5	57	95
PFD (360–760 nm)	232	100	143	100	60	100
PFD-Ultraviolet (360–399 nm)	1.7	0.7	1.6	1.1	0.6	1.0
PFD-Blue (400–519 nm)	65.7	28.4	16.9	11.9	8.2	13.6
PFD-Green (520–559 nm)	33.5	14.5	7.0	4.9	13.5	22.4
PFD-Yellow (560–624 nm)	56.9	24.5	83.6	58.7	27.9	46.4
PFD-Red (625–700 nm)	55.3	23.9	24.3	17.1	7.6	12.7
PFD-Far Red (701–760 nm)	18.7	8.1	9.0	6.4	2.4	3.9
R/FR ratio (660/730 nm) ‡	2.8		2.4		0.1	
DLI (mol m^−2^ d^−1^) ±	10.6/11.7		6.6/7.2		2.9/3.0	

* *PPFD* = photosynthetic photon flux density, *PFD* = photon flux density, *R/FR* ratio = red to far-red ratio, DLI = daily light integral. ** LED = light-emitting diode, HPS = high-pressure sodium lamp, FL = fluorescent light. *** Values represent percentages of total *PFD*. ‡ R/FR ratio is based on the absorption maxima of phytochromes at 660 and 730 nm [47]. ± DLI based on *PPFD/PFD*.

**Table 2:** Effect of three different supplemental lighting systems on the chemical composition of 13 main volatile organic compounds (VOCs) of *Thymus vulgaris* L. cultivated in the greenhouse during fall and winter of Berlin, Germany.

Compound	RI *	Light-Emitting Diode (LED)		High-Pressure Sodium Lamp (HPS)		Fluorescent Light (FL)	
		% **	µg 100 mg^−1^ LDM ***	%	µg 100 mg^−1^ LDM	%	µg 100 mg^−1^ LDM
monoterpene hydrocarbons							
*α*-pinene	938.0 ± 0.4	0.8 ± 0.1	14.03 ± 0.8 ^a^	0.8 ± 0.1	11.41 ± 1.1 ^b^	1.0 ± 0.1	7.22 ± 1.1 ^c^
sabinene	977.8 ± 0.4	1.4 ± 0.4	34.21 ± 8.5 ^a^	1.5 ± 0.6	33.57 ± 6.4 ^a^	1.3 ± 0.5	13.95 ± 1.5 ^b^
myrcene	991.8 ± 0.3	1.6 ± 0.2	35.16 ± 1.8 ^a^	1.5 ± 0.2	28.50 ± 3.6 ^a^	1.8 ± 0.2	17.19 ± 1.6 ^b^
*α*-terpinene	1020.8 ± 0.3	1.9 ± 0.4	39.47 ± 4.3 ^a^	2.1 ± 0.7	33.43 ± 3.2 ^a^	2.2 ± 0.8	19.72 ± 2.1 ^b^
*p*-cymene	1029.1 ± 0.5	8.5 ± 2.2	157.70 ± 27.4 ^a^	8.3 ± 2.5	123.90 ± 8.0 ^a^	6.9 ± 2.5	55.16 ± 4.7 ^b^
limonene	1033.1 ± 0.3	0.6 ± 0.1	9.81 ± 0.7 ^a^	0.6 ± 0.1	8.22 ± 0.8 ^a^	0.6 ± 0.1	4.59 ± 0.5 ^b^
*γ*-terpinene	1064.4 ± 0.8	15.2 ± 5.4	323.20 ± 56.7 ^a^	16.0 ± 4.7	259.90 ± 49.0 ^ab^	20.1 ± 6.3	179.50 ± 22.6 ^b^
oxygenated monoterpenes							
*cis*-sabinene hydrate	1071.7 ± 0.4	1.3 ± 0.1	28.41 ± 2.3 ^a^	1.4 ± 0.2	25.38 ± 1.6 ^a^	1.3 ± 0.3	12.31 ± 0.8 ^b^
linalool	1100.9 ± 0.5	2.6 ± 0.7	61.92 ± 11.3 ^a^	2.7 ± 0.8	52.22 ± 11.2 ^a^	2.3 ± 0.9	21.25 ± 1.9 ^b^
borneol	1173.6 ± 0.3	0.9 ± 0.4	44.44 ± 2.6 ^a^	1.0 ± 0.6	46.50 ± 5.9 ^ab^	1.5 ± 0.7	31.75 ± 3.7 ^b^
thymol	1297.6 ± 1.8	54.6 ± 6.9	1134.00 ± 86.3 ^a^	52.9 ± 6.4	917.10 ± 142.9 ^a^	50.2 ± 7.9	429.90 ± 57.3 ^b^
carvacrol	1304.6 ± 1.1	2.4 ± 0.4	60.82 ± 4.9 ^a^	2.3 ± 0.5	48.82 ± 8.2 ^a^	2.0 ± 0.6	21.42 ± 2.9 ^b^
sesquiterpene hydrocarbons							
*β*-caryophyllene	1436.55 ± 0.5	3.1 ± 1.3	47.69 ± 7.1 ^a^	3.67 ± 1.1	49.50 ± 7.0 ^a^	3.32 ± 1.1	21.91 ± 3.4 ^b^
% of total extract **		94.93 ± 1.50		94.68 ± 2.22		94.53 ± 1.95	
Total VOCs [% g^−1^ LDM] ****		2.7 ± 0.22 ^a^		2.3 ± 0.25 ^a^		1.1 ± 0.10 ^b^	

* Retention indices (RI) relative to C_6_-C_24_ n-alkanes on a HP-5MS column for compound identification. Indices are presented as means ± *SD* with *n* = 192. ** Percentages were calculated from GC-FID TIC data after weight correction and presented as means ± *SD* with *n* = 64. *** Amounts of major compounds were calculated based on density corrected calibration functions obtained from reference standards analyzed under the same GC-FID conditions as the samples. Presented are mean amounts of volatile compounds (µg 100 mg^−1^ L_DM_ (=leaf dry matter)) of four independent spatial replications per light treatment (*n* = 4) ± *SD* of 16 collected dried leaf samples per spatial replication and light treatment (*N* = 192, *n* = 64 dry leaf samples per supplemental light treatment, *n* = 16 dry leaf samples per spatial replication). Significant differences (*p* ≤ 0.05) were determined according to Dunnett’s T3 multiple comparisons test after Brown-Forsythe and Welch ANOVA test (*p* ≤ 0.02). Different letters within a row indicate significant differences at *p* ≤ 0.05, and bold amounts indicate significant differences at *p* ≤ 0.1. **** Percentage of total VOCs (volatile organic compounds) was calculated based on the results of the internal standard (6-methyl-5-penten-2-one), which was co-analyzed in each sample. Presented are mean percentages per g L_DM_ (% g^−1^ L_DM_ (=leaf dry matter)) of four independent spatial replications per light treatment (*n* = 4) ± *SD* of 16 collected dried leaf samples per spatial replication and light treatment (*N* = 192, *n* = 64 dry leaf samples per supplemental light treatment, *n* = 16 dry leaf samples per spatial replication). Significant differences (*p* ≤ 0.05) were determined according to Dunnett’s T3 multiple comparisons test after Brown-Forsythe and Welch ANOVA test (*p* ≤ 0.001). Different letters within a row indicate significant differences at *p* ≤ 0.05 and bold amounts indicate significant differences at *p* ≤ 0.1.

**Table 3:** Fresh and dry plant production as well as content of volatile fraction of thyme (*Thymus vulgaris* L.) per m^2^ under three supplemental lighting systems.

Light Fixture *	FM ** per Square Meter [g m^−2^] ***	DM ** per Square Meter [g m^−2^] ***	VOC ** per Square Meter [mg m^−2^] ****
LED	897.9 ± 64.65^a^	180.2 ± 24.69 ^a^	**2472 ± 626.4 ^a^**
HPS	509.4 ± 72.88 ^b^	102.6 ± 16.87 ^b^	**1273 ± 334.0 ^a^**
FL	158.0 ± 6.73 ^c^	20.62 ± 2.06 ^c^	199.1 ± 30.98 ^b^

* LED = light-emitting diode, HPS = high-pressure sodium lamp, FL = fluorescent light. ** FM = total fresh matter, DM = total dry matter, VOC = total content of volatile organic compounds of total leaf dry matter. *** Presented data are means of cumulated fresh and dry matter productions of four independent spatial replications per light treatment (*n* = 4) ± *SD* of 32 harvested plants per spatial replication and light treatment (*N* = 384, *n* = 128 plants per supplemental light treatment, *n* = 32 plants per spatial replication). Significant differences (*p* ≤ 0.01) were determined according to Dunnett’s T3 multiple comparisons test after Brown-Forsythe and Welch ANOVA test (*p* ≤ 0.001). Different letters indicate significant differences. **** Presented data are means of cumulated volatile productions in thyme leaves of four independent spatial replications per light treatment (*n* = 4) ± *SD* of 16 harvested plants per spatial replication and light treatment (*N* = 192, *n* = 64 dry leaf samples per supplemental light treatment, *n* = 16 dry leaf samples per spatial replication). Significant differences were determined according to Dunnett’s T3 multiple comparisons test after Brown-Forsythe and Welch ANOVA test (*p* ≤ 0.002). Different letters within the column indicate significant differences at *p* ≤ 0.02, and bold amounts indicate a difference by trend at *p* < 0.06.

**Table 4:** Power consumption per square meter of the supplemental lighting fixtures for the production of thyme (*Thymus vulgaris* L.) grown in a greenhouse during fall and winter of Berlin, Germany.

Light Fixture *	Power Consumption per Meter^2^ [W m^−2^]	Power Consumption for Thyme Cultivation [kWh m^−2^]	Power Savings Compared to HPS [%]	Fresh Thyme Production ** [g kWh^−1^ m^−2^]	Dry Thyme Production ** [mg kWh^−1^ m^−2^]	VOC Production *** [mg kWh^−1^ m^−2^]
LED	257.7	454.6	31.3	1.92 ± 0.15 ^a^	396.3 ± 54.31 ^a^	5.4 ± 1.4^a^
HPS	374.9	661.3	*na*	0.77 ± 0.11 ^b^	155.2 ± 25.5 ^b^	1.9 ± 0.5 ^b^
FL	299.4	528.1	20.1	0.30 ± 0.03 ^c^	39.1 ± 3.9 ^c^	0.4 ± 0.1 ^c^

* LED = light-emitting diode, HPS = high-pressure sodium lamp, FL = fluorescent light, *na* = not applicable. ** Presented are calculated average fresh and dry thyme productions per power consumption of each light fixture type within a square meter during the cultivation period (g or mg per kWh and m^2^) of four independent spatial replications per light treatment (*n* = 4) ± *SD* of 32 harvested plants per spatial replication and light treatment (*N* = 384, *n* = 128 plants per supplemental light treatment, *n* = 32 plants per spatial replication). Significant differences (*p* ≤ 0.01) were determined according to Dunnett’s T3 multiple comparisons test after Brown-Forsythe and Welch ANOVA test (*p* ≤ 0.001). Different letters indicate significant differences. *** Presented are calculated average productions of volatile organic compounds per power consumption of each light fixture type within a square meter during the cultivation period (mg per kWh and m^2^) of four independent spatial replications per light treatment (*n* = 4) ± *SD* of 16 harvested plants per spatial replication and light treatment (*N* = 192, *n* = 64 plants per supplemental light treatment, *n* = 16 plants per spatial replication). Significant differences (*p* ≤ 0.05) were determined according to Dunnett’s T3 multiple comparisons test after Brown-Forsythe and Welch ANOVA test (*p* ≤ 0.004). Different letters indicate significant differences.

## Data Availability

The data that support the findings of this study are available from the corresponding author, A.K., upon reasonable request.
